# High frequency of *TERT* promoter mutation in small cell carcinoma of bladder, but not in small cell carcinoma of other origins

**DOI:** 10.1186/s13045-014-0047-7

**Published:** 2014-07-20

**Authors:** Xiaoyong Zheng, Jian Zhuge, Stephania M Bezerra, Sheila F Faraj, Enrico Munari, John T Fallon, Ximing J Yang, Pedram Argani, George J Netto, Minghao Zhong

**Affiliations:** 1 Department of Pathology, Westchester Medical Center/New York Medical College, 100 Woods road, Valhalla 10595, NY, USA; 2 Department of Pathology, Johns Hopkins Hospital, Pathology, Baltimore, MD, USA; 3 Department of Pathology, Northwestern University, Pathology, Chicago, IL, USA

## Abstract

*TERT* promoter mutations were recently discovered in melanoma by next generation sequencing. Subsequently, several malignancies including urothelial carcinoma were also found to be associated with the same *TERT* promoter mutations. Small cell carcinoma (SCC) of the urinary bladder is a rare subtype with an aggressive clinical course. Despite the frequent occurrence of *TERT* promoter mutations in urothelial carcinoma, the incidence of the mutations in SCC of the urinary bladder is unknown. In addition, as a potential molecular marker to distinguish SCC of the urinary bladder from SCC of the prostate, lung (SCLC) and other origins, this information may be clinically useful. We collected a total of 11 cases of SCC of the urinary bladder (10 cases are primary SCC of the urinary bladder; 1 case has primary SCC of the urinary bladder and liver metastasis). We also included 20 cases of SCLC, 2 cases of SCC of the prostate, 5 cases of Merkel cell carcinoma, and 6 cases of SCC from other sites (cervical, GE junction, breast, and soft tissue). In addition, 3 cases of non-neoplastic tissue from the matched SCC of bladder patient and 14 cases of benign urinary bladder were also included. All tumor sections have been examined to confirm the diagnosis and to make sure more than 20% are of tumor content. Genomic DNA was isolated from FFPE tissue and a fragment of the *TERT* promoter (145 bp) was amplified by PCR. The *TERT* promoter mutations are determined by bi-directional Sanger sequencing. All (11/11) SCC of the urinary bladder bear *TERT* promoter mutation C228T. Neither of SCC from all other origins nor matched non-neoplastic tissue contains the *TERT* promoter mutations. We demonstrated a high frequency *TERT* promoter mutation in SCC of the urinary bladder, but not in SCC of other origin, such as the prostate. The findings further illustrate molecular differences between SCC of the urinary bladder and SCC of other origins, despite their shared morphologic and immunophenotypic similarities. The *TERT* promoter mutation may be a biomarker differentiating SCC of the urinary bladder from SCC of other origins.

## Introduction

Small cell carcinoma (SCC) is a distinct clinicopathologic entity that usually originates from the lung but can also arise in almost any extrapulmonary sites. Extrapulmonary small cell carcinomas (ESCCs) are rare, and have been described most frequently in the urinary bladder, prostate, esophagus, stomach, colon and rectum, gallbladder, larynx, salivary glands, cervix, and skin. Small cell carcinoma of the bladder is a rare, aggressive, poorly differentiated neuroendocrine neoplasm that is similar to small cell carcinoma of the lung in clinical behavior. In fact, bladder small cell carcinoma is frequently found in conjunction with conventional urothelial carcinoma and/or other histologic variants: squamous and glandular differentiation. Small cell carcinoma of the prostate is also an unusual and aggressive subtype, only accounting for 0.5–2% of all prostate cancers [[1]]. Approximately 50% of the time, prostatic small cell carcinoma occurs in the background of usual-type acinar carcinoma and the two components may be intermingled, thus suggesting a common origin. These phenomena strongly suggest that different SCC may originate from cells of different primary sites. Disregarding the primary origins however, all SCCs have very similar, if not identical, histopathological morphology and immunophenotype. In addition, small cell carcinoma will occasionally be present with metastatic disease. In this scenario, primary site is difficult to define by tumor morphology and IHC stains.

Small cell carcinoma is currently classified as a discrete entity and the same chemotherapy regimens are used in all small cell carcinoma. Exploring the origin of different SCCs may help us understand the carcinogenesis of this aggressive tumor and contribute to a specific target therapy. Acutely, molecular studies have indicated that small cell carcinoma of urinary bladder and usual types of urothelial cancer are derived from the same clonal population. In addition, up to 50% of small cell carcinoma of prostate carries *ERG* translocation, which is commonly seen only in prostate acinar carcinoma [[2]]. These evidence suggest that at least part of small cell carcinoma of bladder and prostate are not *de novo* and may be derived from usual-types of urothealial carcinoma and prostatic acinar adenocarcinoma, respectively.

Telomerase reverse transcriptase (*TERT*) activities are frequently upregulated in many human cancers, and are thought to be an important mechanism contributing to human tumorigenesis [[3]]. Recently, two studies reported [[4],[5]] a high frequency (approximately 70%) somatic mutation in the *TERT* promoter in melanoma at positions 1,295,228 (C228T) and 1,295,250 (C250T). These mutations create consensus binding motifs for E-twenty six (ETS)/ternary complex factor (TCF) transcription factors (GGA[A/T] or CCGGAA), leading to increased *TERT promoter* activity. Subsequently, theses mutations have been found to be associated with different types of cancer, including urothelial carcinoma, but not in other common carcinoma, such as: lung, prostate, breast and cervical cancers [[6]]. However, there is no report about the mutation status in small cell carcinoma of the bladder, prostate, lung or other origins. In this study, we investigated *TERT* promoter mutations in small cell carcinoma of the bladder, prostate, lung and other primary sites.

## Materials and methods

After IRB (WMC, L-10,884 by New York Medical College) approval, the following cases were collected for this study: 20 cases of SCLC (including 3 metastases); 11 cases of SCC of the urinary bladder (including primary SCC of the urinary bladder from 10 patients, one patient with both primary and liver metastasis), 14 cases of normal urinary bladder tissue. 2 cases of prostatic small cell carcinoma; 5 cases of Merkel cell carcinoma; 2 cases of small cell carcinoma from cervix; 1 case of small cell carcinoma from breast; 1 case of small cell carcinoma from GE junction and 2 cases of small cell carcinoma from soft tissue. *H&E* sections were reviewed by board-certified pathologists to confirm the diagnosis and ensure that ≥20% of the cells used for DNA purification were neoplastic. Genomic DNA from tissues was isolated by using standard procedures of protease K digestion, QIAGEN kit (QIAamp DNA Mini). A fragment of the *TERT* promoter was amplified by polymerase chain reaction (PCR) using primers 5′-CAGCGCTGCCTGAAACTC-3′ (sense) and 5′-GTCCTGCCCCTTCACCTT-3′ (antisense), resulting in a PCR product of 163 bp, which contained the sites of C228T and C250T mutations(chr5: 1,295,228; chr5: 1,295,250, respectively; hg19). Amplification PCR was performed with an initial denaturation at 94°C for 5 min, followed by 40 cycles of 97°C denaturation for 30 sec, 55°C annealing for 30 sec and 68°C elongation for 30 sec. Quality of PCR products was confirmed by gel electrophoresis. Sequencing both strands was performed by using a BigDye terminator v1.1 cycle sequencing kit (Applied Biosystems) on ABI 3500xL genetic analyzer (Applied Biosystems). The statistical analysis was performed by SPSS 16.0 software. Rates of mutation were compared using chi-square test and Fisher’s exact tests among groups. A *P* value less than 0.05 was considered as statistically significant.

## Results

**Figure 1:**
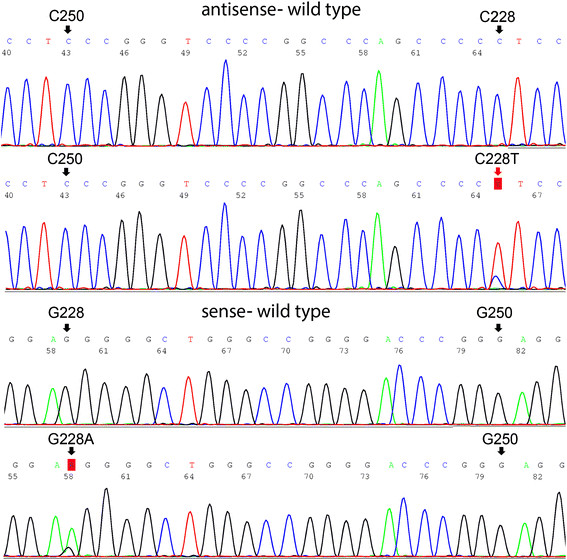
***TERT*promoter mutations in SCC of urinary bladder cancer.** Shown are representative sequence of the wild-type *TERT* promoter and *TERT* promoter mutation as indicated for bladder cancer. The upper portion of the figure shows the sense sequences of the wild-type DNA and the nucleotide changes of C228T mutation. The lower portion of the figure shows the antisense sequences of the wild-type DNA and the nucleotide changes of the G228A mutation.

Among all 11 cases of SCC of the urinary bladder evaluated for *TERT* promoter mutation, 2 were radical cystectomy, 2 were transurethral resections of bladder tumor (TURBT), 5 were bladder tumor biopsy and 1 was with both partial cystectomies and metastatic biopsy from liver. The average patient age was 75 years (range: 53–88), with 6 males and 4 females. Almost all primary cases showed additional non-SCC components including: usual urothelial carcinoma, carcinoma with squamous or glandular differentiation. After PCR and sequencing, *TERT* C228T mutation was identified in all 11 cases (100%) of bladder SCC (Figure 1). The non-neoplastic tissue from 3 cases with separate normal tissue blocks did not show *TERT* promoter mutation (0/3). All cases with benign urinary bladder tissue have no *TERT* promoter mutation (0/14). This confirmed that *TERT* promoter mutation is a high frequency genetic alteration in SCC of bladder and it is somatic. None of SCC of the urinary bladder is GATA-3 positive by immunohistochemistry staining.

In 20 SCLC cases, 3 (15%) were wedge resections, 14 (70%) were lung biopsies, 2 (10%) were metastases liver biopsy, and 1 (5%) was metastasis in adrenal gland. The average patient age was 68 years (range: 56–82), with 9 males and 11 females. None of these cases of SCLC (0/20), including metastases of liver or adrenal, showed no *TERT* promoter mutation.

In 2 prostate small cell carcinomas, one was from prostatic needle biopsy which showed 3 of 12 cores contained SCC; the other one was from a bladder neck lesion biopsy from a patient who had a prostatomy 10 years ago due to cancer. *TERT* promoter mutation was identified in neither case.

In small cell carcinoma from other origins, including 5 cases of Merkel cell carcinoma, 2 case from cervix; 1 case from breast; 1 case from GE junction and 2 cases from soft tissue in extremities, no *TERT* promoter mutation was identified in all of these cases (Table 1).

**Table 1:** ***TERT*promoter mutation in SCC of different origins (Chi-Square Tests*P*** **< 0.05)**

**Location**	***TERT*** **promoter mutation**
SCC of Lung	0% (0/20)
SCC of Bladder	100% (11/11)
Counterpart normal tissue of above case	0% (0/3)
Benign urinary bladder tissue	0% (0/14)
SCC of Prostate	0% (0/2)
Merkel cell carcinoma	0% (0/5)
Other origins (breast, extremities, JE junction, Cervix, etc.)	0% (0/6)

## Discussion

Small cell carcinoma (SCC) most commonly arises in the lower respiratory tract of chronic smokers and is usually rapidly evolving and lethal. It is rare for SCC to arise in extra pulmonary sites which including urinary bladder and prostate. Histologic criteria for the diagnosis of extrapulmonary SCC are the same as those for pulmonary SCC. The tumor usually has a patternless type of diffuse growth, whereas occasionally and focally, nests and trabeculae are observed. The tumor cells have sparse cytoplasm and consequently exhibit nuclear crowding and molding. Nucleoli are often inconspicuous, and the chromatin is finely stippled (salt-and-pepper). Frequent mitoses, crush artifact, geographic necrosis, and Azzopardi effect are seen. In addition to morphological similarity, no immunohistohistochemical (IHC) marker(s) can reliably differentiate SCC among lung, prostate, bladder or other origins. A case series [[7]] in 2001 found TTF-1 positivity in 42% (21 of 50) of extrapulmonary SCC cases, including prostate and urinary bladder (1/3 each) primaries. A series 44 cases of SCC of the urinary bladder cases study also showed: TTF-1+, 11 (25%); synaptophysin+, 22 (50%) [[8]]. TTF-1 is often positive in prostate small cell carcinomas [[9],[10]]. Prostate-specific immunostains (such as PSA, p501s and PSMA) are positive in only a minority of small cell carcinoma (approximately 20–25%) [[10],[11]] AR and NKX3-1 were not expressed in the majority of small cell carcinoma, although the majority of the concurrent acinar foci were positive for these markers [[2]]. Therefore, it is almost impossible for pathologists to determine the origin by SCC by morphology and IHC stains.

Despite their shared histologic morphologies and IHC profiles, some evidence suggest that SCC is not *de novo* and arise from usual type of carcinoma at different primary sites. First of all, SCCs are more frequently admixed with other histologic subtype(s). In fact, almost all SCC of bladder cases in our study demonstrated additional non-SCC components: frequently high grade urothelial carcinoma, as well as squamous and/or glandular differentiation. All SCC of lung cases in this study did not show additional usual carcinoma, perhapsdue to small tissue of biopsy. One of two prostatic SCCs had previous prostatic acinar adenocarcinoma, Gleason score 4 + 3. Additionally, molecular genetic studies have also suggested a common clonal origin for bladder SCC and coexisting bladder UC [[12],[13]]. X chromosomal inactivation analysis [[12]] in females illustrated the same nonrandom inactivation in both SCC of the urinary bladder and usual UC. Identical point mutations of TP53 were found in invasive SCC of the urinary bladder and coexisting UC in situ; further more, no loss of heterozygosity of 9 microsatellite markers and TP53alu was found in either component [[13]]. In prostatic SCC, it has been found that *ERG* rearrangements in nearly half of SCC of the prostate is a similar rate of the same rearrangements in prostatic acinar carcinomas; and these rearrangements have not been detected in SCC of the urinary bladder or lung [[2]]. It has been well established that Merkel cell virus is strongly associated with only Merkel cell carcinoma, but not SCC of any other origins. Recent reports [[14],[15]] also revealed that small cell carcinoma of the ovary, hypercalcemic type, displays frequent inactivating germline and somatic mutations in SMARCA4, and this mutation is very rare in other common tumors. All of these evidence strongly suggest that despite large overlapping of morphology and immunophenotype, SCC from different origins may not have the same oncogenesis at molecular level. Different type of SCC may need specific treatment and management.

*TERT* promoter mutations, originally be discovered in ~70% melanoma, have also been found to be the most common genetic mutations of UC. Interestingly, these mutations have very low incidence in other prevalent carcinomas: lung, prostate and colon cancers. This phenomenon promotes us to evaluate *TERT* promoter mutations in SCC of different origins. Our data showed that no *TERT* promoter mutations were identified in SCC of lung, prostate and other origins, in contrast, all SCC of bladder harbor *TERT* promoter mutation: C228T. These incidences of *TERT* promoter mutations in SCC of different origins are concord with those counterpart usual carcinomas. Multiple studies [[16]–[18]] including our unpublished results demonstrated that up to 70-80% UC carries the *TERT* promoter mutations disregarding grade, stage or location. This study showed 100% SCC of bladder carry *TERT* promoter mutation: C228T. Additionally, no C250T *TERT* promoter mutation, the second most common mutation in UC, is identifies in SCC of bladder. The difference may partly due to relative small number cases (10) of SCC bladder, but the difference is statically significance. To confirm and understand the biological meaning of this difference, larger case number studies are required.

Interestingly, the tumors from both primary and liver metastasis of SCC bladder demonstrated the same mutation. This further supports that *TERT* promoter mutations can be used as a SCC bladder marker to determine the primary site of metastatic SCC. Since low frequency to none of *TERT* promoter mutations are in SCC of lung, prostate and other sites, only positive mutation could be suggestive of bladder origin. The negative mutations could be unfavored bladder origin, but could not be used to distinguish among other origins. The studies of using TERT IHC stain as a surrogate test for *TERT* promoter mutations are few and results are controversial. TERT expression upregulation is very common in many tumors and probably is caused by different molecular mechanisms. These mutations create consensus binding motifs for E-twenty six (ETS)/ternary complex factor (TCF) transcription factors (GGA[A/T] or CCGGAA) leading to increased *TERT* promoter activity and subsequently *TERT* transcriptional upregulation. Presumably, TERT IHC stain can detect TERT expression upregulation. However, TERT expression upregulation could not be distinguished between *TERT* promoter mutations and other molecular mechanisms, such as gene amplification. *TERT* promoter mutations in urothelial bladder cancer are not associated with TERT mRNA levels [[17]]. Thus, molecular method is still the only reliable approach to detect *TERT* promoter mutations.

In summary, we reported high frequency *TERT* promoter mutation in SCC of bladder, but not in SCC of other origins. This further illustrated the heterogeneity of SCC at molecular level and provided an addition piece of evidence that origination of SCC bladder may be as the same as that of usual urothelial carcinoma. Most importantly, *TERT* promoter mutation can be used as a potential biomarker for bladder small cell carcinoma.

## Competing interests

The authors declare that they have no competing interests.

## Authors' contribution

XZ, JF and MZ designed study; XZ, JZ and MZ performed study; SB, SF, EM XY, PA and GN contributed important reagent and material; XZ and MZ wrote manuscript. All authors read and approved the final manuscript.
